# Supplementary material for: The effect of three different pre-match warm-up structures on male professional soccer players’ physical fitness
Source: PeerJ. 2023 Aug 3;11:e15803. doi: 10.7717/peerj.15803 (PMC10404391; doi:10.7717/peerj.15803)
Supplement: File S1 [file peerj-11-15803-s002.docx]

| **Supplementary Table .**  **Phases and exercises for warm-ups** | | |
| --- | --- | --- |
| **Phase** | **Subphase** | **Exercises** |
| *Aerobic phase* | Jogging (4 min) | Jogging around the field |
|  | Running and mobility (4 min) – two rows of 15 m | Running  Skipping  Heel lift  Side run  Back stroke  Zigzag  Zigzag backwards  Turn to the right  Turn to the left  Push with shoulder |
|  | Dynamic stretching (2 min) | Hamstring  Quadriceps  Adductors  Abductors  Calves |
| *Neuromuscular Phase* | Strengthening (2 min) | Half-squat bilateral 5 reps  Half-squat unilateral 5 reps  Side step 5 reps |
|  | Balance (2 min) | Bilateral jumps and receptions 5 reps  Unilateral jumps and receptions 5 reps |
| *Velocity Phase* | Velocity over 5 and 10 m (2 min) | 2 sprints with 30 sec of rest (linear)  2 sprints with 30 sec of rest (non-linear) |
| *Ball-specific phase* | Passes (4 min) | Pairs: passes in several distances (from 5 to 35 m) |
|  | Small-sided game (4 min) | 5 vs. 5 20x20 m, 2 sets of 1 min 30 sec with 1 min of rest |
